# Validity of fractional exhaled nitric oxide and small airway lung function measured by IOS in the diagnosis of cough variant asthma in preschool children with chronic cough

**DOI:** 10.1186/s13223-023-00835-x

**Published:** 2023-09-09

**Authors:** Ying Hu, Shouyan Zheng, Zhiqiang Chen, Dan Yu, Tianxia Lai, Yao Chen, Wei Liao

**Affiliations:** Department of Pediatrics, The First Affiliated Hospital of Army Medical University, 30 Gaotanyanzheng Street, Shapingba District, Chongqing, 400038 China

**Keywords:** Chronic cough, Cough variant asthma, Fractional concentration of exhaled nitric oxide, Preschool children, Small airway lung function

## Abstract

**Background:**

To investigate the role of combined impulse oscillometry (IOS) and fractional exhaled nitric oxide (FeNO) in the diagnosis of cough variant asthma (CVA) in preschool children.

**Methods:**

A total of 197 preschool-aged children with chronic cough were selected from the paediatric outpatient clinic. Allergy histories were collected for all children along with IOS and FeNO. Paediatric respiratory specialists divided the children into a CVA group (n = 90) and a noncough variant asthma (nCVA) group (n = 107) according to the diagnostic criteria for CVA After diagnostic treatment, the correlation between the FeNO and IOS values and the diagnosis in the two groups was analysed, and the area under the curve (AUC) of each index was calculated.

**Results:**

(1) X5 was significantly different between the CVA group and the nCVA group (− 4.22 vs. − 3.64, p < 0.001), as was the FeNO value (29.07 vs. 16.64, p < 0.001). (2) Receiver operating characteristic (ROC) analysis showed that the AUCs of FeNO alone and X5 alone were 0.779 and 0.657, respectively, while the AUC of FeNO (cut-off value of 18 ppb) plus X5 (cut-off value of -4.15 cmH2O/(l/s)) reached 0.809.

**Conclusions:**

Children with CVA may have small airway dysfunction at an early stage. For preschool children with chronic cough, the combination of FeNO and X5 can better identify those with CVA.

*Trial registration number:* This trial was registered with and approved by the Chinese Clinical Trial Registry, with registration number ChiCTRcRRC-17011738, and was reviewed and approved by the Ethics Committee of Southwest Hospital.

## Background

Cough is one of the most common symptoms of respiratory diseases in children. The Guideline for the Diagnosis and Treatment of Chronic Cough in Chinese Children defines chronic cough as coughing with a disease course of more than 4 weeks in children [1]. A national multicentre study published in 2012 and the Clinical Practice Guide for Diagnosis and Treatment of Cough in Children in China issued by the Respiratory Group of the Pediatric Branch of Chinese Medical Association in 2021 have both noted that cough variant asthma (CVA) is the most common cause of chronic cough in Chinese children, especially preschool and school-age children, accounting for 40% of chronic cough in the latter [1–3]. Some asthma patients have significantly decreased lung function, but cough remains the only or main symptom. Some typical asthma patients may have transient wheezing symptoms, but persistent cough is their main symptom. These two conditions, also known as cough-predominant asthma (CPA), have become important clinical concerns in recent years. The 2020 version of the American College of Chest Physicians (ACCP) Cough Guidelines collectively referred to cough-variant asthma (CVA) and CPA as "chronic cough due to asthma" [4], and in the 2020 version of the European Respiratory Society (ERS) Cough Guidelines, they were referred to as "asthmatic cough" [5].

CVA is a special type of childhood asthma, most often starting before school age and manifesting as an airway inflammatory disease. CVA patients have only cough (no other symptoms), airway dysfunction (variable obstruction & AHR), and response to Rx.. In 2019, the Global Initiative for Asthma (GINA) did not discuss the criteria for the diagnosis of CVA, instead suggesting that it should be determined according to the airway hyperresponsiveness (AHR) value and treatment response [6]. Lung function impariment in asthmatic children often starts at preschool age; therefore, the guidelines emphasize that asthma treatment should start as early as possible and that complete control should be achieved to reduce the occurrence of adult chronic obstructive pulmonary disease (COPD) [7]. However, due to the young age and poor understanding of preschool children, the challenge test cannot be performed, and auxiliary examinations for preschool children's CVA are limited. Therefore, it is necessary to find an objective, accurate and noninvasive method suitable for preschool children that can diagnose CVA at an early stage.

Asthma is an airway hyperresponsive inflammatory disease characterized by small airway obstruction. Cough variant asthma is a prodrome of asthma. Increasing evidence shows that small airway dysfunction can exist independently of any effects on the large airway [8] and can occur before clinical symptoms and pulmonary function impariment [9]. Therefore, whether small airway dysfunction occurs and its probability in CVA is an important issue of clinical concern. Currently, the most commonly used clinical small airway function assessment standard is the forced expiratory flow (FEF) between 25 and 75% of the forced vital capacity (FVC), also known as FEF25-75. However, preschool children cannot actively participate in the forced expiration lung function test. The emergence of another lung function examination technology, impulse oscillometry (IOS), solves this problem. This examination only requires quiet breathing and does not require an unusual amount cooperation from children, and therefore, any child older than 3 years of age can complete it. Moreover, studies have found that IOS indices reactance area (AX), reactance of the respiratory system at 5 Hz (X5), resistance of the respiratory system at 5 Hz (R5) and 20 Hz (R20), the difference between R5 and R20 (R5-R20) and the resonant frequency (Fres) can appropriately reflect small airway function [10, 11].

The fractional concentration of exhaled nitric oxide (FeNO) is a biomarker recommended by the American Thoracic Society (ATS) [12] for airway inflammation, especially airway eosinophilic inflammation, and It can assess the inflammation of both the central airway and small airways [13]. Recent meta-analyses have shown that FeNO has important value in the diagnosis of CVA in adult patients [14, 15]. Chen et al. [16] reported the diagnostic value of FeNO plus small airway function indices for CVA in adult patients with chronic cough. FeNO alone [17] and FeNO plus the small airway function parameter maximum expiratory flow 50% (MEF50%) have high efficacy in the diagnosis of CVA in school-age children with chronic cough [18]. The latest Chinese guidelines also point out that for children with chronic cough suspected of CVA, the FeNO test should be used to assist in diagnosis [3]. However, the diagnostic efficacy of FeNO for CVA in preschool children still lacks sufficient clinical evidence, and the diagnostic value of FeNO plus IOS indices (such as X5, Z5, R5, R20 and Fres) for preschool children with CVA has never been studied. Therefore, this study selected IOS and FeNO measurements suitable for preschool children and explored the probability of small airway dysfunction in CVA and the value of these measurements in diagnosing CVA in preschool children with chronic cough through a cross-sectional investigation.

## Methods

### Study subjects

A total of 197 preschool children with chronic cough (longer than 4 weeks) who visited the Pediatric Outpatient Department of the First Affiliated Hospital of Army Medical University from July 2019 to January 2020 were included. All patients consented to participate.

The inclusion criteria were as follows:

(1) age 3 to 6 years, regardless of sex; (2) cough course longer than 4 weeks; (3) no abnormalities on chest X-ray or other imaging and no bronchodilator, oral or inhaled corticosteroid, anti-allergy drug and other drug use history within 3 days; (4) no history of upper respiratory tract infection, fever or sore throat within 4 weeks.

The exclusion criteria were as follows:

(1) Chronic respiratory disorders, such as cystic fibrosis, tuberculosis, congenital bronchopulmonary dysplasia and classical asthma; and (2) other complications, including epilepsy, congenital heart disease, congenital respiratory disease and thoracic deformity.

The CVA diagnostic criteria were as follows:

Chronic cough and CVA were diagnosed according to the recommendations of the Chinese National Guidelines on the diagnosis and management of cough [1].

(1) Continuously coughing for more than 4 weeks, usually dry cough, often occurring at night and/or in the morning. Coughing worsens after exercise or exposure to cold air, with no clinical signs of infection or ineffective treatment with antibiotics over a long period of time; (2) Significant relief of cough symptoms after diagnostic treatment with bronchodilators; (3) Pulmonary ventilation function is normal, and bronchial provocation test indicates airway hyperresponsiveness; (4) Have a history of allergic diseases and a positive family history of allergic diseases. A positive allergen test can assist in diagnosis; (5) Excluding chronic cough caused by other diseases.

However, due to the age and characteristics of preschool children, the bronchial challenge test could not be completed. Therefore, diagnostic treatment (aerosolized bronchodilator for 2 weeks plus inhaled corticosteroids (ICS) for 2 weeks) [1, 4, 5] was applied, and a respiratory specialist later evaluated whether the diagnostic treatment was sufficiently effective to confirm the diagnosis of CVA.

According to the above criteria, the children with chronic cough (n = 197) were divided into a CVA group (n = 90) and a noncough variant asthma (nCVA) group (n = 107).

### FeNO measurement

A Nano coulomb nitric oxide analyser (Sunvou-P100, China) was used to measure FeNO according to the FeNO standardized measurement guidelines provided by the American Thoracic Society (ATS)/ ERS [19]. The measurement was repeated 3 times, and the average value was used as the final measurement result.

### Measurement of IOS indices

The IOS indices, including R5, R20, Z5, X5 and Fres, were measured using an impulse oscillometer (Jaeger, Germany) in accordance with the ERS standard [20].

### Sample number and statistical methods

#### Sample number

The observation index used in this study to calculate the sample size was the FeNO value, based on the study of Fang et al. [21] The sample size was calculated using the test of superiority comparing two-sample means and was designed according to a two-group parallel-controlled randomized block. The formula is as follows:

$$N = \frac{{(Z_{1 - \alpha } + Z_{1 - \beta } )^{2} \times (\sigma_{1}^{2} + \sigma_{2}^{2} )}}{{(\mu_{1} - \mu_{2} - \delta )^{2} }}$$

In this study, *α* = 0.05 (two-sided), *β* = 0.20, $\mu_{1} = 24.{12}$, $\mu_{2} = 16.35$, $\sigma_{1}^{2} = {14}{\text{.47}}^{2}$ and $\sigma_{2}^{2} = 12.{01}^{2}$. If the allowable error δ = 20.97 is known in advance, the sample size of each group is calculated to be 75 cases. Considering that there are many factors that can affect the clinical trial process, the sample size can be appropriately increased. The number of cases in each group with this scheme was therefore set as approximately 90.

### SPSS 18.0 software was used for the statistical analysis of all data

The measurement data in this study conformed to a normal distribution and are represented as the mean ± standard deviation ($\overline{x} + s$). The t test was used for comparisons between groups. Counting data are expressed as the rate (%), and the chi-square test was used for comparisons between groups. The receiver-operating characteristic (ROC) curve and area under the curve (AUC) were used to analyse the diagnostic sensitivity and specificity of FeNO and small airway indices for CVA. Two-sided P values of < 0.05 were considered statistically significant.

## Results

### Comparison of basic clinical characteristics between the two groups of children

Statistical analysis of the basic information of the two groups of children: Differences in age, height and weight between the two groups of children were not statistically significant (P > 0.05) and thus were comparable (Table 1).

**Table 1:** Comparison of basic clinical characteristics between the CVA group and nCVA group ($\overline{x} + s$)

	CVA group	nCVA group	P
Sex (male/female)	46/44	64/43	0.220
Age (years)	3.82 ± 0.57	3.91 ± 0.97	0.436
Height (cm)	102.02 ± 4.95	101.59 ± 4.27	0.511
Weight (kg)	16.24 ± 2.14	16.08 ± 2.36	0.612
BMI	15.57 ± 1.44	15.54 ± 1.71	0.876
history(y/n)	49/41	52/55	0.413

Demographic parameters, atopy and blood eosinophil counts of the study subjects history: Allergy history/family history of allergies: Patients with previous allergic diseases (eczema/allergic rhinitis, etc.) or a family history of allergies according to the collected history.

### Comparison of the differences in FeNO and the IOS small airway function indices X5, Z5, R5, R20 and Fres between the two groups

**Fig. 1:**
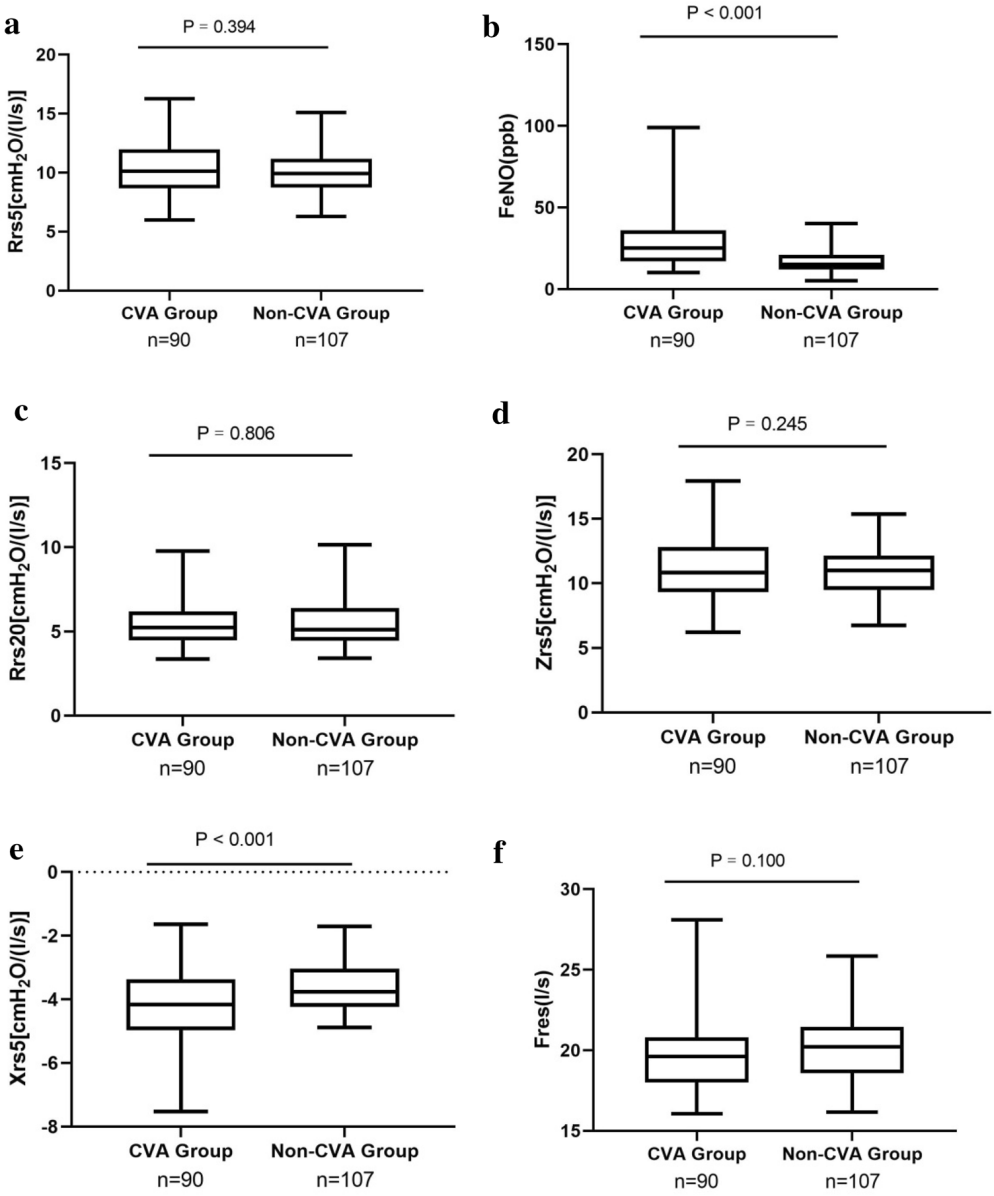
Comparison of FeNO and IOS small airway function indices between CVA group and nCVA group. **a** Comparison of R5 between CVA group and nCVA group. **b** Comparison of FeNO between CVA group and nCVA group. **c** Comparison of R20 between CVA group and nCVA group. **d** Comparison of Z5 between CVA group and nCVA group. **e** Comparison of X5 between CVA group and nCVA group. **f** Comparison of Fres between CVA group and nCVA group

The FeNO values and IOS small airway function index X5 were significantly different between the CVA group and the nCVA group. The results are shown in Table 2. As shown in Fig. 1, the FeNO and X5 values of the CVA group were significantly higher than those of the nCVA group (Fig. 1a and d). Z5, R5, R20, and Fres were not significantly different between the two groups (Fig. 1b, c, e and f).

**Table 2:** Comparison of FeNO and IOS indices between the CVA group and nCVA group ($\overline{x} + s$)

Index	CVA group	nCVA group	P
FeNO (ppb)	29.07 ± 15.56	16.64 ± 7.15	< 0.001
Z5 [cmH_2_O/(l/s)]	11.22 ± 2.47	10.85 ± 1.96	0.245
R5 [cmH_2_O/(l/s)]	10.31 ± 2.26	10.06 ± 1.86	0.394
X5 [cmH_2_O/(l/s)]	− 4.22 ± 1.12	− 3.64 ± 0.78	< 0.001
R20 [cmH_2_O/(l/s)]	5.48 ± 1.35	5.53 ± 1.39	0.806
Fres (l/s)	19.68 ± 2.17	20.18 ± 2.08	0.100
AX (cmH_2_O/l)	33.90 ± 12.76	31.84 ± 10.12	0.207

FeNO (ppb), Z5 [cmH_2_O/(l/s)], R5 [cmH_2_O/(l/s)], R20 [cmH_2_O/(l/s)], X5 [cmH_2_O/(l/s)], Fres (l/s) and AX (cmH_2_O/l)

### FeNO plus the IOS index X5 has greater diagnostic value for CVA in preschool-aged children

**Fig. 2:**
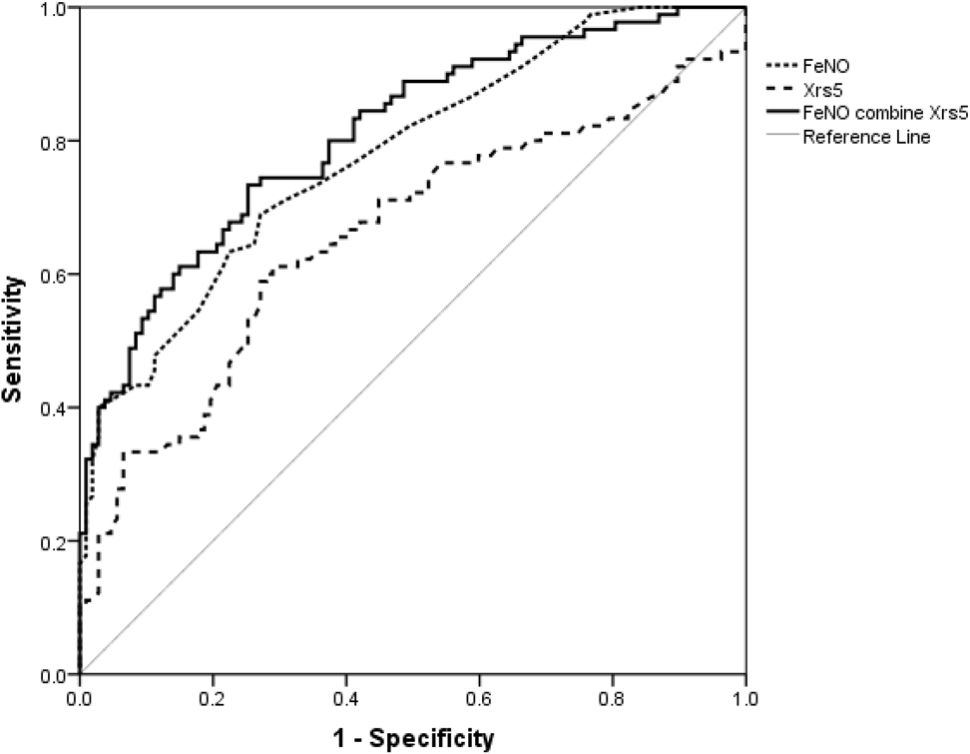
Comparison of the AUCs of X5 alone, FeNO alone and FeNO plus X5

When FeNO alone was used for the ROC analysis to assess asthma, the AUC was 0.779. When the IOS index X5 alone was used, the AUC reached 0.657. FeNO (18 ppb) plus X5 (-4.15 cmH2O/(l/s)) achieved a higher AUC than X5 alone (0.809). The results suggest that X5 can assess asthma well and that FeNO plus X5 performs even better (Fig. 2 and Tables 3 and 4).

**Table 3:** AUCs of IOS indices alone, FeNO alone and FeNO + X5

Variables	AUC
FeNO	0.779
X5	0.657
FeNO + X5	0.809

**Table 4:** Comparison of the AUC of X5 alone, FeNO alone and FeNO + X5

	Cut-off value(s)	Sensitivity	Specificity	Youden index
X5 [cmH_2_O/(l/s)]	− 3.30	0.611	0.710	0.321
FeNO (ppb)	19.50	0.689	0.729	0.418
X5 [cmH_2_O/(l/s)] + FeNO (ppb)	− 4.15, 18.00	0.733	0.748	0.481

## Discussion

Studies have shown that the main aetiologies of chronic cough in children are CVA, upper airway cough syndrome, respiratory tract infections and gastroesophageal reflux [1, 22]. NAEB is uncommon in (Chinese) children. It may be difficult to diagnose NAEB due to the need to obtain induced sputum from children, which is difficult and risky in children..The prevalence of chronic cough is high among preschool children, but due to age limitations, it is hard to evaluate using the bronchial challenge test. Therefore, there is an urgent need for an objective, accurate and noninvasive method for diagnosing CVA that is more suitable for preschool children.

CVA is a special type of childhood asthma with a nature and pathogenesis similar to those of allergic asthma and is characterized by idiosyncrasies, airway hyperresponsiveness and chronic airway inflammation [23]. FeNO is a biomarker that reflects airway inflammation, especially airway eosinophilic inflammation, of both the central and peripheral airways and even the alveoli [13]. Published research has shown that compared with patients with other types of chronic cough, the FeNO values of patients with CVA are significantly higher [14]. However, due to differences in the optimal threshold and diagnostic sensitivity and specificity of FeNO, its value in diagnosing CVA is controversial [15, 17]. In this study, ROC curve analysis was used to analyse the accuracy of FeNO in diagnosing CVA. When the threshold value was 18.5 ppb, the diagnostic sensitivity was 68.9%, the specificity was 72.9%, and the area under the curve was 77.9%. This shows that FeNO has fair diagnostic value for CVA and that it can play an important role in clinical practice for differentiating and assisting in the diagnosis of CVA. This is consistent with the research conclusion of Chen et al. [24].

Asthma is an airway inflammatory disease. In adults and older children, a large amount of attention is given to the large airway structure, but the majority of the small airway is often ignored. Although the GINA guidelines emphasize multi-indicator methods in the description and management of asthma, in general, clinical attention has not yet been paid to children's small airways. Small airways are those with a diameter of less than 2 mm and no cartilage; in healthy adults, small airway resistance only accounts for 10% to 20% of the total airway resistance. However, in healthy infants and young children, this fraction can reach 50% to 90% and is the main source of airway obstruction in asthma. In the past 20 years, the small airways were once called the silent zone. However, now it is recognized that small airway disease is not silent, but the early state of the disease that has not been effectively detected. Increasing evidence shows that small airway dysfunction can exist independently of the influence of the large airway, that it can occur before clinical symptoms and pulmonary function damage, and that it is closely related to asthma control, acute attack and prognosis. CVA is a special asthma clinical phenotype, and previous studies have found that 40% of CVA can develop into typical asthma [25]. Yi et al. [26] also found that more than half of CVA patients had small airway dysfunction. Therefore, small airway dysfunction may be an important and relatively neglected phenotype of CVA, and the evaluation of small airway function is particularly important when assessing asthma in preschool children. Due to the characteristics of young children, the most appropriate method for evaluating small airway function is still noninvasive lung function testing. Although FEF25-75 is commonly used in the clinic to assess small airway function, some studies have shown that MEF50 can reflect small airway dysfunction in children with CVA [18]. Because preschool children cannot actively cooperate, the application of these indices is limited. IOS examination only requires the subject to breathe normally; no special active cooperation is needed. IOS indices at different frequencies including R5-R20, AX, X5 and Fres can reflect the resistance of different parts of the respiratory system and therefore small airway function [9–11]. These indices can be used to evaluate asthma control well and predict future asthma attacks [26–28]. This study shows that the X5 value of the CVA group was significantly higher than that of the nCVA group. In the IOS examination, X5 indicates the reactance at 5 Hz, which represents the elastic resistance of the lung. Shin et al. [29] found that the IOS lung function index X5 can accurately reflect severity (intermittent or continuous) relative to symptom improvement, medication use and oral corticosteroid use. Our previous research also found that X5 could be used to reflect asthma control in preschool children [27].

Zhu's studies have shown that the FeNO level is significantly negatively correlated with the lung function indices FEF25-75, MEF50 and MEF25, which reflect small airway function [18]. The results of this study showed that the FeNO level is positively correlated with the small airway function index X5 (absolute value), suggesting that small airway dysfunction may be related to airway inflammation. Liu et al. [30] reported that FeNO plus IOS had increased sensitivity and specificity in the diagnosis of small airway dysfunction in adult asthma patients. A recent report showed that FeNO plus MMEF or MEF50 demonstrated significantly elevated sensitivity and specificity in the diagnosis of CVA in school-aged children [18].

The current study showed that in preschool children with CVA, compared with FeNO alone, the combination of FeNO and IOS small airway indices can increase diagnostic sensitivity. In particular, FeNO plus X5 leads to an increased AUC (0.809), which represents an increase in the sensitivity and specificity in diagnosing CVA. These results indicate that FeNO is a reflection more of the degree of airway eosinophilic inflammation than of small-airway obstruction. When combined with IOS small airway indices, such as X5, FeNO can better reflect the degree of small-airway inflammation. Therefore, this study suggests that the combination of FeNO and the IOS index X5 can better diagnose CVA in preschool children at cut-off values of 18 ppb and -4.15, respectively.

## Conclusions

The combination of FeNO (cut-off value of 18 ppb) and the IOS index X5 (cut-off value of -4.15) can reach an AUC of 80.9% in diagnosing CVA in preschool children. Simultaneous FeNO and IOS examination is conducive to the early treatment of CVA in preschool children with chronic cough.

### Limitations of the study

1. Since the IOS small airway indicators AX and R5-R20 have no normal reference values, the incidence of small airway abnormalities was not calculated.
2. This study is a single-centre clinical trial with a limited number of enrolled cases. More convincing results could be obtained with a multicentre study involving a larger sample size.

## Data Availability

The data that support the findings of this study are available from the corresponding author upon reasonable request.

## Abbreviations

IOS: Impulse oscillometry
FeNO: Fractional expired nitric oxide
CVA: Cough variant asthma
GINA: Global Initiative for Asthma
AUC: Area under the curve
ROC: Receiver operating characteristic
FEF25-75: Forced expiratory flow at 25–75% of forced vital capacity
FVC: Forced vital capacity
FEV1: Forced expiratory flow at 1s
FEF50: Forced expiratory flow at 50% of forced vital capacity
R5-R20: Measurement of respiratory resistance between R5 and R20
AX: Area of reactance
X5: Represents the reactance throughout the respiratory system
RV: Residual volume
TLC: Total lung capacity
ICS: Inhaled corticosteroid
CalvNO: Calculated alveolar nitric oxide
FEVz: Z score for FEV 1
sRawz: Z score for specific airway conductance
MMEFz: Z score for mid-maximal expiratory flow
AIT: Allergy immunotherapy
LABA: Long-acting beta-agonist
LAMA: Long-acting muscarinic antagonist
ACT: Asthma control test scores
CASI: Composite asthma severity index
SAD: Small airway disease (dysfunction)
